# A visualization analysis of Traditional Chinese Medicine for influenza prevention and treatment: advances, hotspots, and future trends

**DOI:** 10.3389/fmed.2026.1852941

**Published:** 2026-06-23

**Authors:** Chang Sun, Qi Chen, Yang Wang, Shuojing Yuan, Ying Su, Le Zhang

**Affiliations:** College of Basic Medical Sciences, Changchun University of Chinese Medicine, Changchun, China

**Keywords:** advances, bibliometrics, future trends, hotspots, influenza, traditional Chinese medicine, visualization analysis

## Abstract

**Objectives:**

As an acute respiratory infectious disease, influenza continues to impose a substantial public health burden worldwide. This study aims to systematically review the progress of research on the treatment of influenza with Traditional Chinese Medicine (TCM) from 2005 to 2025, identify current research hotspots, and forecast future development trends, in order to provide a clear and systematic reference framework for subsequent research.

**Methods:**

A bibliometric and scientometric analysis was conducted using the Web of Science Core Collection (WOSCC), PubMed, and Scopus databases. Following the PRISMA 2020 guidelines, the retrieved records underwent a comprehensive deduplication process and stringent quality control checks. By comprehensively applying CiteSpace, VOSviewer, and the R-based Bibliometrix package, metrics and visualization were performed across multiple dimensions, including publication volume, geographical contribution, annual trends, national/regional influence, core authors and institutions, and keywords.

**Results:**

A total of 1,527 publications were included in this study. Since 2014, publication output in this field has shown significant growth, with a rapid upward trend emerging after 2020. At the national and institutional level, China ranked first globally in both the number of publications and total citation frequency. Research institutions in China not only serve as the dominant force in this field but also act as hubs for international collaboration. Notable contributions were made by institutions such as the Chinese Academy of Sciences, Beijing University of Chinese Medicine, and the China Academy of Chinese Medical Sciences. Journal analysis revealed that the Journal of Ethnopharmacology is the most influential journal in this domain. In terms of scholarly impact, Yang Zifeng ranked first in both h-index and publication output, establishing them as the most prolific and influential core scholar in the field. Keyword analysis indicated that research focuses on core themes such as “herbal medicine” and “antiviral activity.” The evolutionary trajectory demonstrates a shift from traditional clinical practice toward modern mechanistic investigation. Driven by emerging public health events such as COVID-19, the field has rapidly integrated cutting-edge methodologies like network pharmacology, reflecting distinct characteristics of contemporary responsiveness and interdisciplinary convergence.

**Conclusion:**

This analysis confirms that TCM for influenza has matured into a structured and interdisciplinary research field. Substantial evidence supports its multi-component and multi-target therapeutic model as a clinically effective strategy against influenza. Future efforts should prioritize the integration of mechanistic insights with standardized clinical translation to enhance global antiviral preparedness.

## 1 Introduction

Influenza (Flu) is an acute respiratory infectious disease caused by the influenza virus, characterized by a high incidence rate, and significant disease burden (1, 2). According to data from the WHO, approximately one billion people are infected with influenza globally each year, of whom 3–5 million cases progress to severe illness, resulting in an estimated 290,000–650,000 respiratory-related deaths annually. Influenza infection frequently leads to severe lung injury and can even trigger secondary bacterial pneumonia, thereby inducing acute lung injury (ALI) and multiple organ failure, posing a critical threat to critically ill patients (3–5). The primary challenge in influenza prevention and control lies in the virus’s strong mutability, particularly antigenic drift and shift. These phenomena not only gradually diminish the protective efficacy of vaccines but also readily induce antiviral drug resistance (6). Taking the widely used neuraminidase inhibitors, such as oseltamivir, as an example, their clinical application already faces certain limitations. Furthermore, the WHO’s 2024 clinical practice guidelines for influenza have adopted a more cautious stance regarding antiviral therapy for non-severe cases (7). These realities underscore the urgent need for safer, more effective, and sustainable intervention strategies for influenza prevention and treatment.

Against this backdrop, TCM, characterized by its holistic regulation, multi-target intervention, and syndrome differentiation-based treatment, has garnered increasing attention in the prevention and control of respiratory infectious diseases, particularly influenza. With a long-standing history of clinical application and a wealth of practical experience, TCM has demonstrated distinct advantages not only in preventing infection, alleviating symptoms, and promoting recovery, but also in reducing the risk of progression to severe disease and improving overall patient outcomes (8). In recent years, relevant research has evolved from evaluating the clinical efficacy of single herbs, herbal formulas, and proprietary Chinese medicines to elucidating molecular mechanisms involving active ingredients, signaling pathways, and immunomodulation, highlighting the substantial research and application value of TCM in combating influenza (9–11). Therefore, systematically reviewing the current status and developmental trends of TCM in treating influenza is of great significance for consolidating existing achievements and identifying future directions.

However, despite the growing volume of research in this field, existing literature remains fragmented regarding research hotspots, core authors and institutions, collaboration networks, and knowledge evolution pathways, lacking a systematic overarching overview. To address this gap, the present study employs bibliometric methods to conduct a comprehensive analysis of the literature on TCM treatment for influenza. We systematically examined publication trends, countries/regions, institutions, authors, journals, and keywords, while integrating co-citation analysis to identify research hotspots and emerging frontiers (12). This study aims to delineate the evolutionary trajectory, core themes, and unresolved issues within this domain, thereby providing references for subsequent studies, clinical trial design, and strategic research planning, as well as offering data support for the further translation and application of TCM in influenza prevention and control.

## 2 Methods

### 2.1 Databases and search strategy

This study adhered to the PRISMA 2020 Statement for the systematic identification and screening of relevant publications. The literature search was conducted in the WOSCC, Scopus, and PubMed databases. Specifically, the WOSCC, recognized for its rigorous academic selection criteria and comprehensive citation network, holds high authority in medicine and life sciences. The search was confined to its Science Citation Index Expanded (SCIE) and Emerging Sources Citation Index (ESCI) editions to ensure literature representativeness and continuity. The Scopus database features broad, interdisciplinary coverage, effectively supplementing journals and conference proceedings not indexed in Web of Science, which is particularly valuable for capturing cross-disciplinary research trends. PubMed, a biomedical literature database maintained by the U.S. National Library of Medicine, is a key source for accessing highly relevant medical literature, serving to enhance the precision of the search. By integrating these three databases, the retrieval aimed to maximize literature coverage and systematically reveal the research landscape of the field.

The search strategy was constructed around the core themes of “TCM” and “influenza,” employing a combination of subject headings and free-text terms. Boolean operators (AND, OR) and wildcard symbols (*) were used to form the search queries, covering different expressions of related terminology. To improve accuracy, the search in WOSCC was limited to the TS field, in Scopus to the TITLE-ABS-KEY field, and in PubMed to the Title/Abstract field. TCM-related terminology = (“traditional Chinese medicine” OR “patent herbal drug” OR “herbal medication” OR “Chinese patent medicine” OR “herbal formulas” OR “herbal extract” OR “Chinese herbal preparation” OR “Chinese herbal medicine” OR “herbal medicine” OR “traditional herbal medicine” OR “traditional medicine” OR “Chinese herbal decoction”). Influenza-related terminology = (“influenza” OR “flu” OR “infectious cold” OR “viral cold” OR “seasonal influenza” OR “influenza A virus” OR “influenza B virus” OR “influenza C virus” OR “influenza D virus” OR “haemophilus influenzae”). The search strategies applied to each database are summarized in Table 1. All retrieval and data export procedures were completed on 27 January 2026, to ensure data consistency and reproducibility. The exported records were saved in txt format, containing core metadata fields such as article title, authors, publication year, journal name, keywords, abstract, and citation counts.

**TABLE 1:** Database-specific search strings and Boolean combinations.

Databases	Search strategies
WOSCC	TS = (“influenza” OR “flu” OR “infectious cold” OR “viral cold” OR “seasonal influenza” OR “influenza A virus” OR “influenza B virus” OR “influenza C virus” OR “influenza D virus” OR “haemophilus influenzaeavian”) AND TS = (“traditional Chinese medicine” OR “patent herbal drug” OR “herbal medication” OR “Chinese patent medicine” OR “herbal formulas” OR “herbal extract” OR “Chinese herbal preparation” OR “Chinese herbal medicine” OR “herbal medicine” OR “traditional herbal medicine” OR “traditional medicine” OR “Chinese herbal decoction”)
Scopus	(TITLE-ABS-KEY (“influenza” OR “flu” OR “infectious cold” OR “viral cold” OR “seasonal influenza” OR “influenza A virus” OR “influenza B virus” OR “influenza C virus” OR “influenza D virus” OR “haemophilus influenzaeavian”) AND TITLE-ABS-KEY(“traditional Chinese medicine” OR “patent herbal drug” OR “herbal medication” OR “Chinese patent medicine” OR “herbal formulas” OR “herbal extract” OR “Chinese herbal preparation” OR “Chinese herbal medicine” OR “herbal medicine” OR “traditional herbal medicine” OR “traditional medicine” OR “Chinese herbal decoction”)) AND ((PUBYEAR AFT 2004) AND (PUBYEAR BEF 2026))
PubMed	(“influenza”[Title/Abstract] OR “flu”[Title/Abstract] OR “infectious cold”[Title/Abstract] OR “viral cold”[Title/Abstract] OR “seasonal influenza”[Title/Abstract] OR “influenza A virus”[Title/Abstract] OR “influenza B virus”[Title/Abstract] OR “influenza C virus”[Title/Abstract] OR “influenza D virus”[Title/Abstract] OR “haemophilus influenzaeavian”[Title/Abstract]) AND (“traditional Chinese medicine”[Title/Abstract] OR “patent herbal drug”[Title/Abstract] OR “herbal medication”[Title/Abstract] OR “Chinese patent medicine”[Title/Abstract] OR “herbal formulas”[Title/Abstract] OR “herbal extract”[Title/Abstract] OR “Chinese herbal preparation”[Title/Abstract] OR “Chinese herbal medicine”[Title/Abstract] OR “herbal medicine”[Title/Abstract] OR “traditional herbal medicine”[Title/Abstract] OR “traditional medicine”[Title/Abstract] OR “Chinese herbal decoction”[Title/Abstract])

### 2.2 Data screening and management

Following the preliminary search, a total of 875 records were retrieved from the WOSCC, 1,453 from Scopus, and 604 from PubMed. Two investigators (Sun and Chen) independently screened all retrieved records for relevance. Any disagreements arising during the screening process were resolved by a third investigator (Su), who made the final decision. After excluding irrelevant studies, the full metadata—including titles, authors, affiliations, countries, publication years, abstracts, keywords, and references—were exported in both plaintext and CSV formats. To unify the data structure, Python (version 3.11) was utilized to convert the Scopus CSV files into the plaintext format (Full Record and Cited References) consistent with WOSCC and PubMed. Data cleaning was also performed using Python (version 3.11). The main steps included: removing duplicates based on DOIs; deleting “retracted” publications and records with “Anonymous” authors; eliminating virtual institutions; and merging and standardizing duplicate institution names.

Inclusion Criteria: (1) Document type: Publicly accessible articles and reviews; (2) Research scope: Studies focusing on TCM treatment of influenza, including human clinical studies, animal models, and cellular mechanism research; (3) Language: English-language publications exclusively; (4) Timeframe: Publication date between 1 January 2005 and 31 December 2025.

Exclusion Criteria: (1) Document type: Non-academic records (e.g., editorials, letters, conference abstracts, retractions, corrections); (2) Research scope: Studies unrelated to TCM treatment of influenza, or those limited to phytochemical extraction and isolated *in vitro* pharmacological assays devoid of mechanistic investigation; (3) Data integrity: Records with inaccessible full texts or substantial metadata deficiency.

**FIGURE 1:**
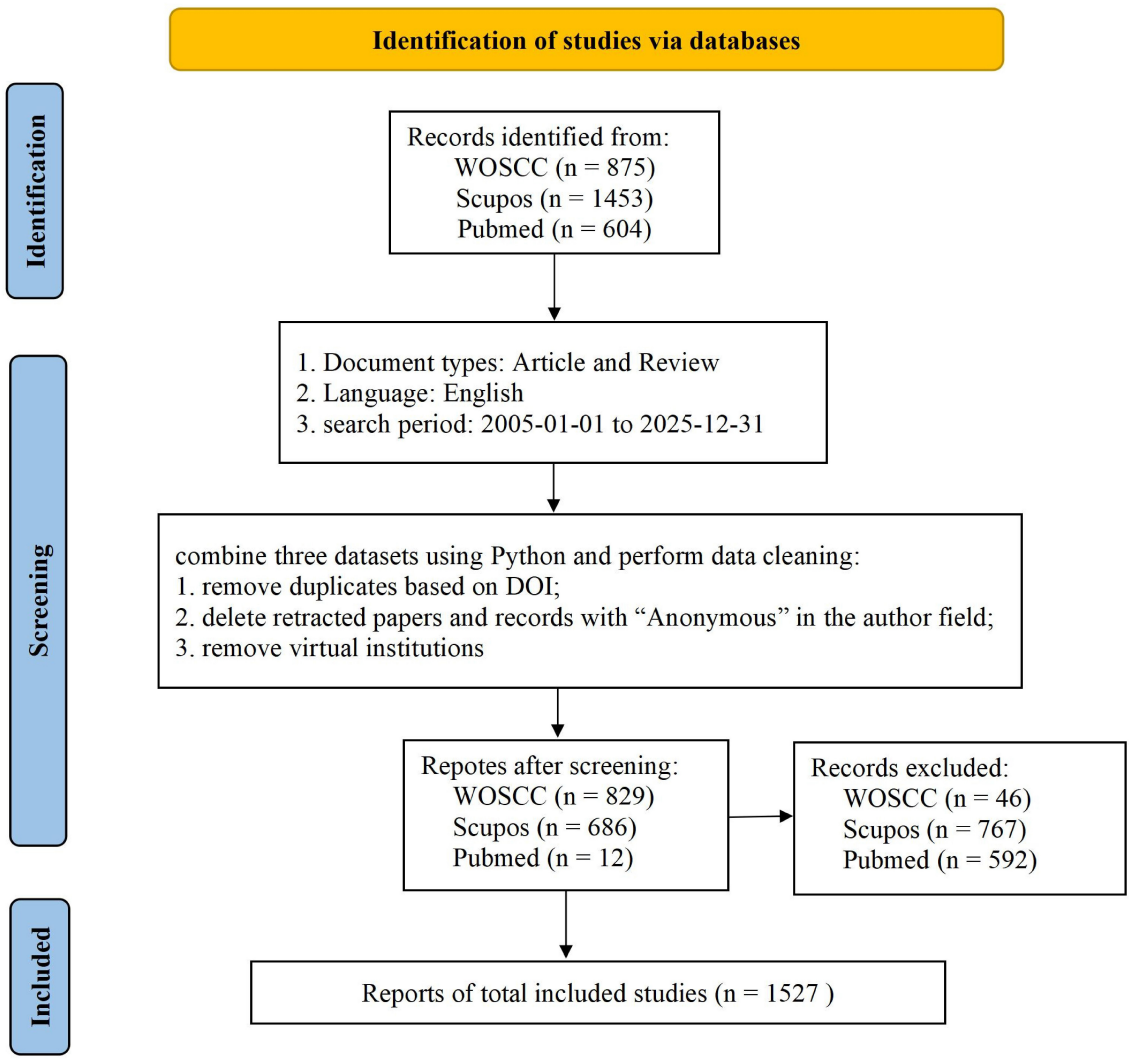
The flowchart for literature search, selection.

A total of 1,527 publications met the inclusion criteria and were incorporated into the bibliometric analysis. Of these, 829 originated from WOSCC, 686 from Scopus, and 18 from PubMed. The PRISMA flow diagram detailing the search and screening process is illustrated in Figure 1.

### 2.3 Data analysis and visualization software

The data contain no identifiable patient information; therefore, no ethical review was required. Data analysis was performed using CiteSpace (version 6.4.R1), VOSviewer (version 1.6.20), and Bibliometrix (version 4.4.3).

#### 2.3.1 CiteSpace

The data analysis in this study was performed using CiteSpace (version 6.4.R1) (13). The dataset covered the period from January 2005 to December 2025. The time span was divided into annual time slices. The node types selected for analysis were authors, institutions, and keywords. Author and institution names were standardized before analysis by unifying full names and abbreviations. To improve network clarity and visualization, the Pathfinder, pruning sliced networks, and pruning the merged network methods were applied. Knowledge maps were then generated to visualize the associations and structural relationships among researchers, institutions, and keywords.

#### 2.3.2 VOSviewer

Co-occurrence analysis was performed using VOSviewer (version 1.6.20) developed by the Centre for Science and Technology Studies at Leiden University (14). The analysis included authors, institutions, and keywords as node types. Minimum occurrence or citation thresholds were applied to filter out low-frequency items, thereby reducing network complexity and improving visualization. Cluster and network visualizations were generated using the default VOSviewer normalization and layout settings.

#### 2.3.3 Bibliometrix

Bibliometrix^1^ is an open-source bibliometric analysis package developed in the R language (15). It was co-created by Dr. Massimo Aria and Professor Corrado Cuccurullo and is primarily used for comprehensive bibliometric analyses, including the calculation of basic indicators such as publication output, author/institution/country productivity, journal distribution, g-index, h-index, citation counts (NC), and number of publications (NP), as well as relational network analysis and the mapping of scientific knowledge structures (16, 17).

## 3 Results

### 3.1 Annual publication trends

**FIGURE 2:**
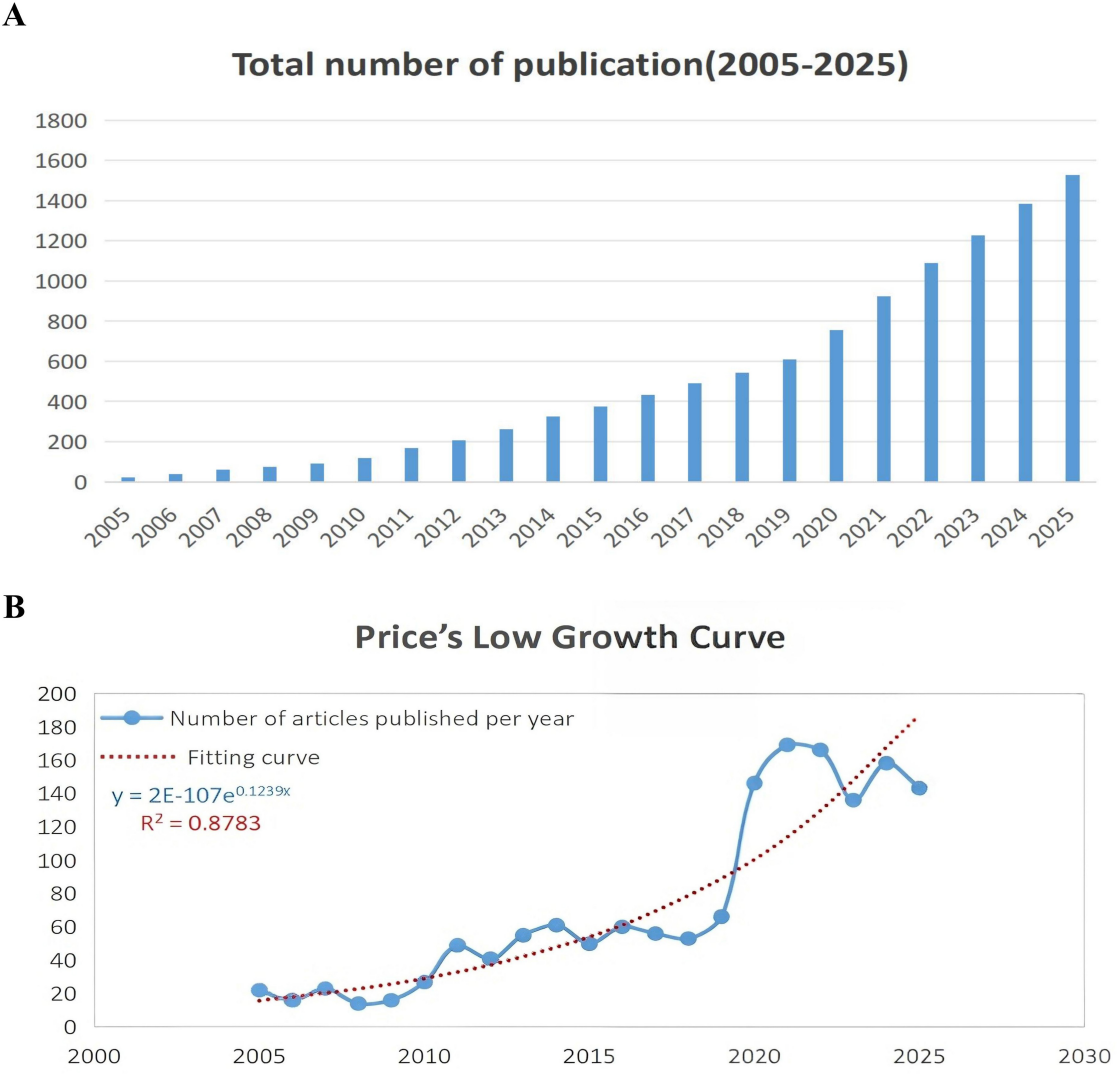
Annual publication trend analysis (2005–2025). **(A)** Cumulative number of publications from 2005 to 2025, illustrating the development trajectory of the field. **(B)** Price’s Law curve fitting analysis of cumulative publications, revealing an exponential growth pattern in research on TCM for influenza treatment.

Figure 2A illustrates the temporal growth trajectory of cumulative publications in the field of Traditional Chinese Medicine for influenza treatment from 2005 to 2025, demonstrating a pronounced exponential growth pattern with an average annual growth rate of 9.81%. Based on variations in the growth trend, this period can be divided into three distinct phases: (1) Steady Growth Phase (2005–2013): During this period, the annual publication output grew slowly, indicating that the field was still in its early developmental stage. In 2008, only 14 publications were recorded, reflecting relatively low research activity. (2) Accelerated Growth Phase (2014–2019): The number of publications showed a consistent and noticeable upward trend, with an increasing growth rate, marking the entry of the field into a phase of steady expansion. (3) Rapid Growth Phase (2020–2025): Beginning in 2020, annual publication output exhibited explosive growth, entering a period of high productivity. The peak was reached in 2021 with 169 publications, indicating a sharp rise in research interest. To further quantify this growth pattern, Figure 2B presents a fitting analysis of the cumulative publication growth curve based on Price’s law. The resulting model equation is y = 2E-107e^0.1239x^, with an R^2^ = 0.8783, indicating a high goodness-of-fit between the model and the observed data. This result confirms that research in the field of Traditional Chinese Medicine for influenza treatment has entered a period of rapid development characterized by exponential growth, suggesting that the research scale, academic influence, and scientific investment in this field will continue to expand.

### 3.2 Country/region distribution

**FIGURE 3:**
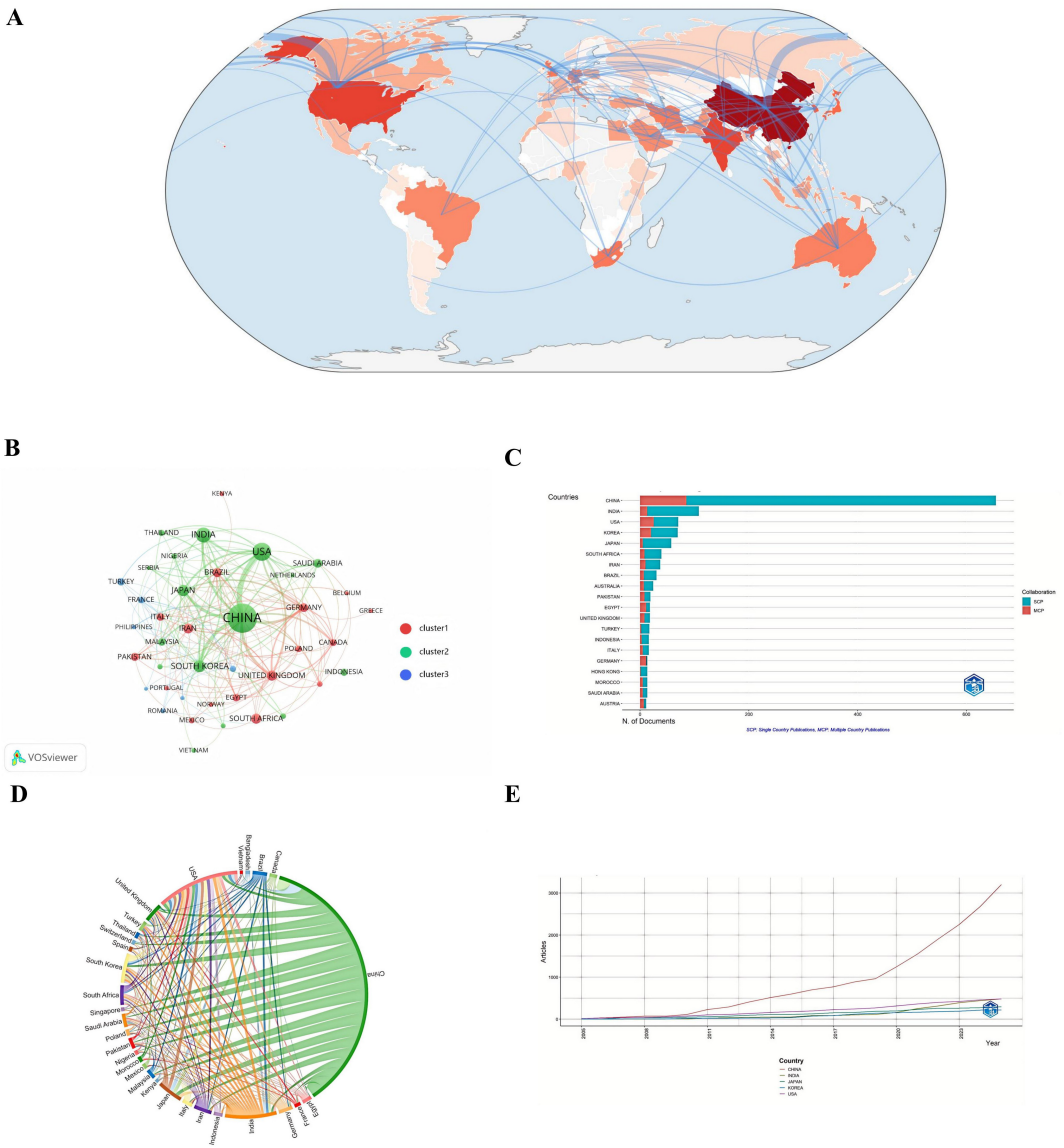
Global trends and collaboration networks in publications (2005–2025). **(A)** Countries’ collaboration map. The intensity of blue shading signifies higher collaboration rates, while the thickness of the connecting lines shows the strength of collaboration between countries. **(B)** Country clustering analysis. Nodes represent countries or regions, with their size proportional to the publication output. The thickness of the edges indicates the strength of co-authorship links between countries. Colors of the nodes show collaboration clusters found by the layout algorithm, where countries in the same color group have more frequent collaborations. Spatial closeness indicates stronger ties. **(C)** Leading countries by number of studies and collaboration type. Stacked horizontal bars display each country’s publication count, partitioned into single-country publications (SCP) and multiple-country publications (MCP). Teal segments denote SCP and salmon/red segments denote MCP (legend “Collaboration” in the panel). The MCP proportion indicates the extent of international collaboration. **(D)** Network map of national research output and cooperative relations (chord diagram). Every outer arc represents a country or region, with its length indicating the total collaborative output of that country within the network. Ribbons connect pairs of countries; ribbon width represents the strength of their collaboration (number of co-authored documents). Ribbon colors follow the color of the originating arc. **(E)** Top five countries/regions by publications. Lines show the cumulative number of publications from 2005 to 2025.

As shown in Figures 3A,B,D, the analysis of the global collaboration network in the research field of Traditional Chinese Medicine for influenza treatment reveals a closely connected network centered on China, linking Asia, Europe, and North America. These charts demonstrate close collaborations between China and countries such as the United States, Japan, Canada, Australia, the United Kingdom, and Germany, reflecting an extensive pattern of international cooperation. In Figures 3C,E, the country/region distribution in the field of Traditional Chinese Medicine for influenza research highlights China’s prominent global leadership, not only in terms of the highest number of published papers but also the highest citation count. India, the United States, and Japan follow in terms of publication output. Notably, China entered a phase of rapid growth after 2011, which turned into explosive expansion after 2020, far surpassing the relatively flat linear growth curves of countries such as the United States, India, Japan, and South Korea, and establishing a lead by orders of magnitude. Overall, the data underscore the continued expansion of the global research network. The concentration of research output in regions such as Asia, Europe, and North America indicates that the use of Traditional Chinese Medicine in influenza treatment has attracted widespread attention from researchers worldwide.

### 3.3 Institutional distribution

**FIGURE 4:**
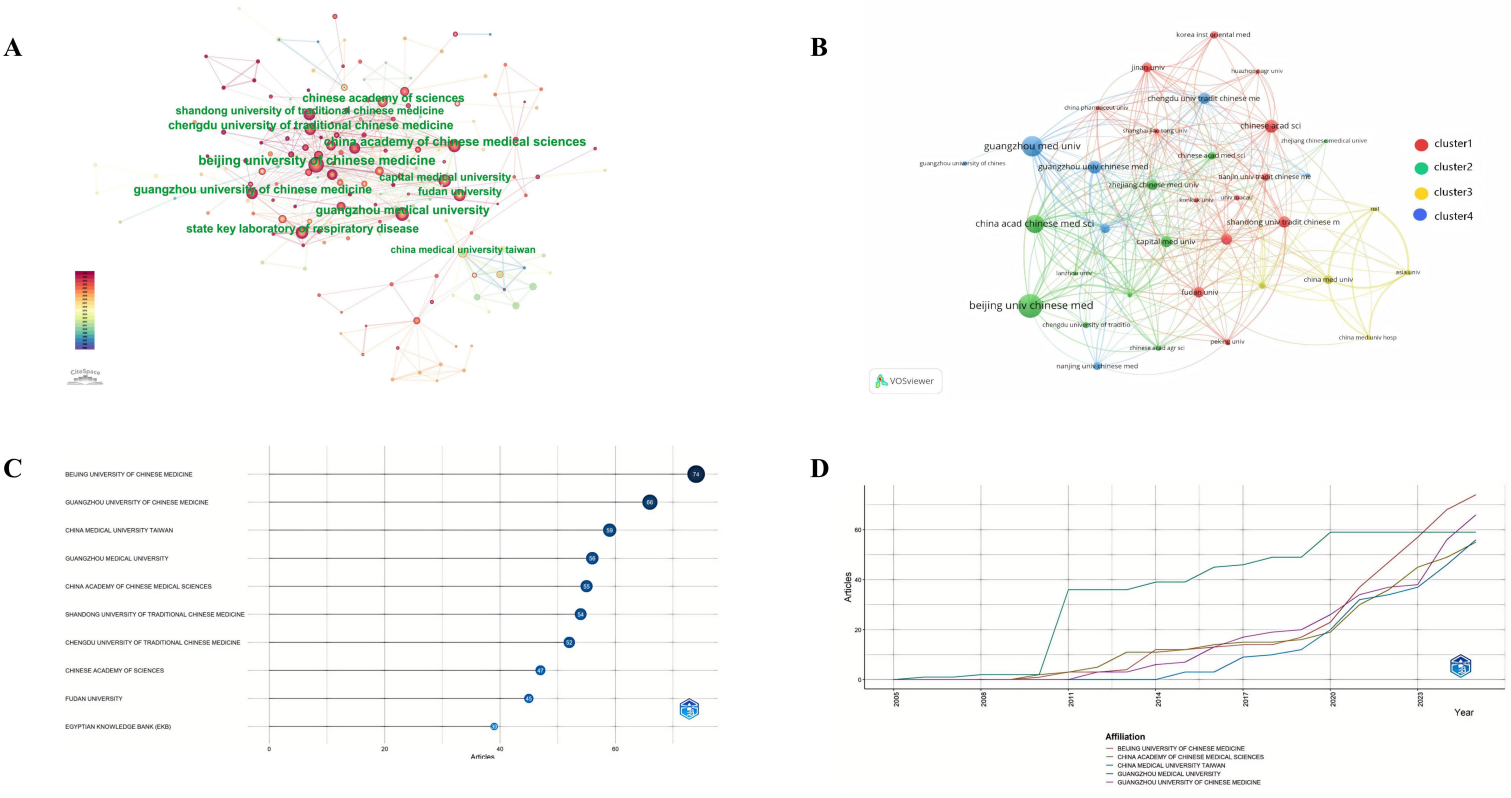
Institutional contributions and collaboration networks in the field of TCM for influenza treatment. **(A)** Research institution network. Nodes symbolize institutions, with their size showing the volume of publications and the thickness of lines representing the strength of collaborations. **(B)** Institutional collaboration network. Node size reflects the amount of collaboration activity, and unique colors signify clusters, often indicating regional or institutional affiliations. Line thickness reflects the strength of collaboration, as indicated by the count of shared publications. **(C)** Top 10 institutions by citation impact. **(D)** Institutional publication trends. The x-axis represents years, while the y-axis shows the number of publications.

Figures 4A,B reveal that research in the field of TCM for influenza treatment has formed a closely interconnected collaboration network centered around universities and research institutes. Institutions such as the Chinese Academy of Sciences, Beijing University of Chinese Medicine, China Academy of Chinese Medical Sciences, and Guangzhou University of Chinese Medicine occupy central positions in the network, characterized by larger nodes and dense connecting lines. Collaboration extends beyond Traditional Chinese Medicine universities to include comprehensive universities (e.g., Fudan University), medical universities (e.g., Capital Medical University), specialized hospitals (e.g., Peking Union Medical College Hospital), and local institutions, forming an interdisciplinary and cross-regional research collaboration system. Scientific output in this field shows a concentration of resources and achievements. As illustrated in Figure 4C, Beijing University of Chinese Medicine and Guangzhou University of Chinese Medicine lead significantly in publication volume, constituting the first tier and serving as the primary contributors to knowledge production. This aligns with their central roles in the collaboration network. Trend analysis of annual publications in Figure 4D indicates that output from major institutions generally accelerated after 2017, marking the entry of the field into a golden period of expansive growth. These results demonstrate that Chinese institutions not only dominate research on TCM for influenza treatment but also act as central hubs in its collaborative network. Although a well-established domestic collaboration network has been formed, international cooperation remains relatively limited, which may constrain further deepening and innovative breakthroughs in the field.

### 3.4 Author contributions

**FIGURE 5:**
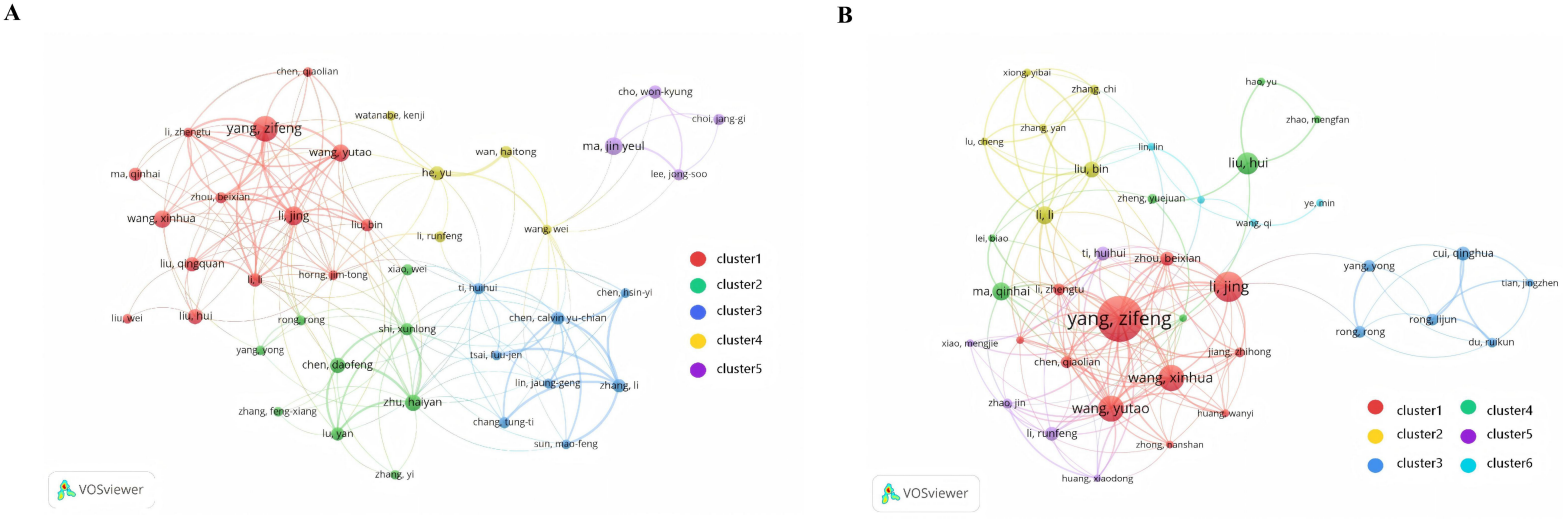
Co-authorship and co-cited authorship networks in the field of TCM for influenza treatment (2000–2025). **(A)** Co-authorship network. Nodes represent authors, sized according to publication counts. Colors indicate author clusters. **(B)** Co-cited authorship network: The thickness of the edges between nodes, which are linked by collaborative publications, reflects the number of joint works. Colors indicate clusters that represent research groups.

Between 2005 and 2025, research on the treatment of influenza with Traditional Chinese Medicine exhibited a highly collaborative academic landscape. A total of 8,274 authors contributed to 1,527 publications. Among them, only 62 authors published independently, with an average of 6.5 co-authors per paper, reflecting the extensive collaborative patterns prevalent in this multidisciplinary field (Table 2). Figures 5A,B further elucidate the collaboration patterns and author influence. Yang Zifeng emerged as the most prolific and influential author in the field, with an h-index of 15 from 25 publications and a cumulative total of 1,261 citations, demonstrating sustained and profound academic impact. While both Li Jing and Ma Jin-yuel have an h-index of 10, an integrated analysis of their g-index and m-index suggests that the former exhibits higher academic efficiency, whereas the latter is recognized for more landmark representative work. Notably, the paper “Honeysuckle-encoded atypical microRNA2911 directly targets influenza A viruses,” to which Li Jing contributed, received the highest average annual citation count (30.25), indicating its enduring scholarly influence (18). The co-citation author network in Figure 5B highlights the collective influence of these leading authors. Yang Zifeng, Li Jing, and Ma Jin-yuel, as core figures, are closely interconnected through a dense co-authorship network, demonstrating their pivotal roles in advancing the research. The research community in this field exhibits a clear hierarchical distribution, ranging from established core contributors (e.g., Yang Zifeng) to rapidly emerging new researchers (e.g., Chen Daofeng). Moreover, the majority of scholars have a g-index higher than their h-index, indicating that the research quality in this field is generally high and tends to produce influential core publications.

**TABLE 2:** Top 10 authors in the field of Traditional Chinese Medicine (TCM) for influenza treatment.

Author	h_index	g_index	m_index	TC	NP	PY_start
Yang Zifeng	15	25	1.25	1,261	25	2015
Li Jing	10	15	0.833	675	15	2015
Ma Jinyeul	10	14	0.714	280	14	2013
Wang Yutao	9	14	0.75	472	14	2015
Zhu Haiyan	9	12	0.692	301	12	2014
Chen Calvin Yu-chian	8	9	0.5	258	9	2011
Chen Daofeng	8	11	0.889	276	11	2018
Cho Won-kyung	8	9	0.571	215	9	2013
Wang Xinhua	8	11	0.8	140	13	2017
Li Zhengtu	7	7	0.583	211	7	2015

TC, total citations; NP, number of publications; PY_start, year of first publication. The 10 authors were automatically ranked by Bibliometrix based on the corresponding bibliometric indicators.

### 3.5 Journals and co-journals

**FIGURE 6:**
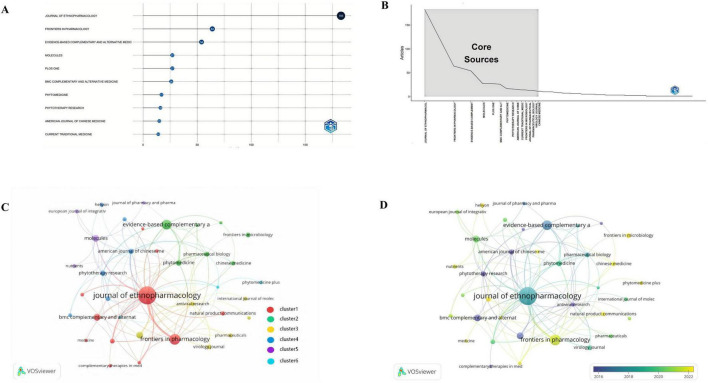
Comprehensive analysis of relevant journals and citation networks in the field of TCM for influenza treatment (2005–2025). **(A)** Top journals by publication count: The x-axis represents article numbers, while the y-axis represents journals. **(B)** Bradford’s Law analysis: Recognizes essential journals based on how publications are distributed. **(C)** Journal citation network. Each node represents a journal. Node size is proportional to the number of publications, while links indicate citation relationships between journals. Line thickness reflects the strength of citation connections. Distinct colors denote clusters of journals that frequently cite each other, corresponding to thematic areas of research. **(D)** Journal co-citation network: Each node symbolizes a journal, with its size based on how often it is co-cited. The links show co-citation connections, where thicker lines denote stronger co-citation ties. Colors highlight clusters of journals frequently co-cited together.

Figure 6A shows that the Journal of Ethnopharmacology is the most influential journal in the research field of TCM for influenza treatment, having published 183 related articles, highlighting its central role in disseminating high-impact research. Following closely, Frontiers in Pharmacology (64 articles) and Evidence-Based Complementary and Alternative Medicine (54 articles) have also made significant contributions to the development of the field. Table 3 further validates their academic influence through bibliometric indicators: Journal of Ethnopharmacology ranks first with an h-index of 54, and its 183 articles have been cited 9,120 times cumulatively, demonstrating outstanding per-article impact. Located in the JCR Q1 category, it is widely recognized as a cornerstone journal and the primary knowledge producer in this domain. In contrast, Phytotherapy Research, with only 16 articles, has received 1,756 citations, making it the leader in per-article influence. The influence of these journals is reflected not only in their publication volume but also profoundly in their academic standing and guiding role.

**TABLE 3:** Top 10 most influential journals in the field of Traditional Chinese Medicine (TCM) for treating influenza.

Source	h_index	g_index	m_index	TC	NP	PY_start	IF	JCR
Journal of Ethnopharmacology	54	89	2.455	9,120	183	2005	5.4	Q1
Frontiers in Pharmacology	24	44	2.182	2,014	64	2016	4.8	Q1
Evidence-Based Complementary and Alternative Medicine	21	40	1.105	1,689	54	2008	2.65	Q3
BMC Complementary and Alternative Medicine	20	26	1	1,309	26	2007	3.8	Q2
PLOS One	19	27	1.188	964	27	2011	2.6	Q1
Molecules	14	27	0.824	920	27	2010	4.6	Q2
Phytotherapy Research	13	16	0.591	1,756	16	2005	6.3	Q1
American Journal of Chinese Medicine	12	15	0.545	445	15	2005	5.5	Q1
Journal of Pharmaceutical and Biomedical Analysis	9	13	0.643	308	13	2013	3.1	Q2
Pharmaceutical Biology	9	13	0.429	628	13	2006	4.8	Q1

IF, impact factor; JCR, journal citation reports. The 10 most influential journals were automatically ranked by bibliometrix based on the corresponding bibliometric indicators.

Figure 6B reveals the distribution of core journals in this field based on Bradford’s Law. The results indicate that the Journal of Ethnopharmacology plays a predominant role, serving as the most significant source of knowledge output. Journals such as Frontiers in Pharmacology and Evidence-Based Complementary and Alternative Medicine are situated within the segment of the curve showing a steep decline, collectively forming a concentrated cluster of core contributing journals. Figure 6C further illustrates the trend of concentration among high-impact sources, demonstrating that the aforementioned three journals dominate both in terms of publication volume and academic influence. The co-citation network analysis in Figure 6D highlights the close scholarly connections among these core journals. Their patterns of being cited together confirm stable collaborative and knowledge exchange relationships within the scientific community.

### 3.6 References and articles

Between 2005 and 2025, the review dataset on TCM for influenza included 1,527 articles from 586 different sources, supported by 45,157 references, with an average of 32.27 citations per article. The relevant studies primarily focused on the antiviral activity and immunomodulatory effects of various classic anti-influenza Chinese medicinal materials, single compounds, and compound prescriptions, with particular attention to the molecular mechanisms involving key inflammatory signaling pathways such as TLR3, NF-κB, and MAPK.

**FIGURE 7:**
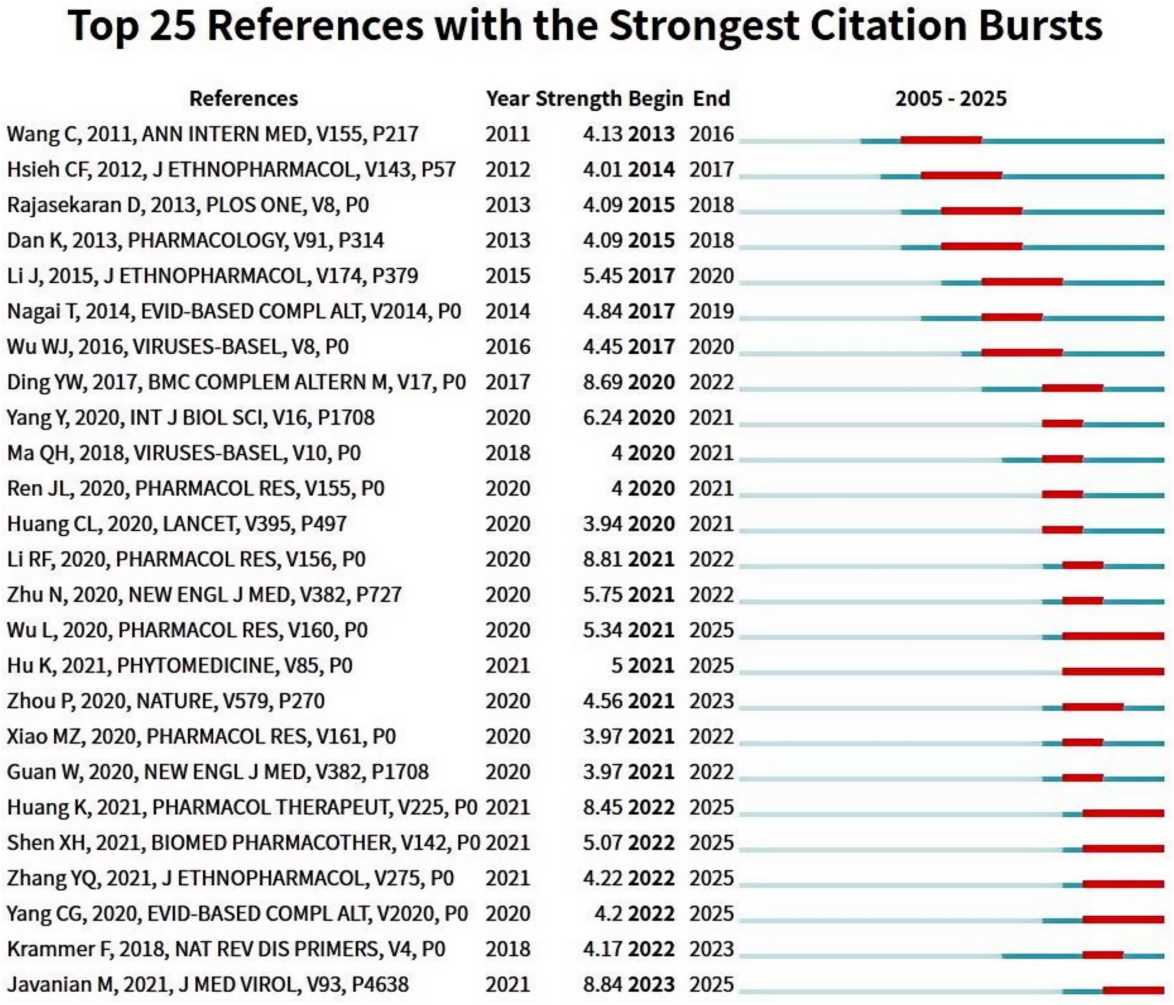
Top 25 references with the strongest citation bursts.

Figure 7 presents the 25 most influential references in research on the treatment of influenza with Traditional Chinese Medicine, ranked by their citation burst strength between 2005 and 2025. It reveals key publications that suddenly attracted high attention during specific periods, leading or defining research trends. For example, the study by Javanian et al. stood out with a citation burst strength of 8.84 in 2023 (19), reflecting heightened scholarly interest during that period in the etiology, epidemiology, transmission, clinical manifestations, and prevention/control of influenza. Based on the citation burst characteristics, the research trajectory in this field can be divided into three distinct phases: Early Clinical and Mechanistic Exploration Phase (2011–2017): The literature mainly focuses on clinical efficacy validation of traditional Chinese medicines for influenza, such as Maxing Shigan Decoction, as well as basic pharmacological mechanism studies of Chinese medicinal materials (20–25). Mid-phase Deepening and Platform Phase (2018–2019): The number of burst publications decreased, and research deepened into basic sciences such as virology and immunology (26). COVID-19-Driven Research Burst Phase (2020–2025): Beginning in 2020, a large number of highly intense and long-lasting citation bursts emerged, with research focus rapidly shifting toward the prevention and treatment of COVID-19 with Traditional Chinese Medicine, forming a new peak of academic attention (27–32).

### 3.7 Keywords analysis

**FIGURE 8:**
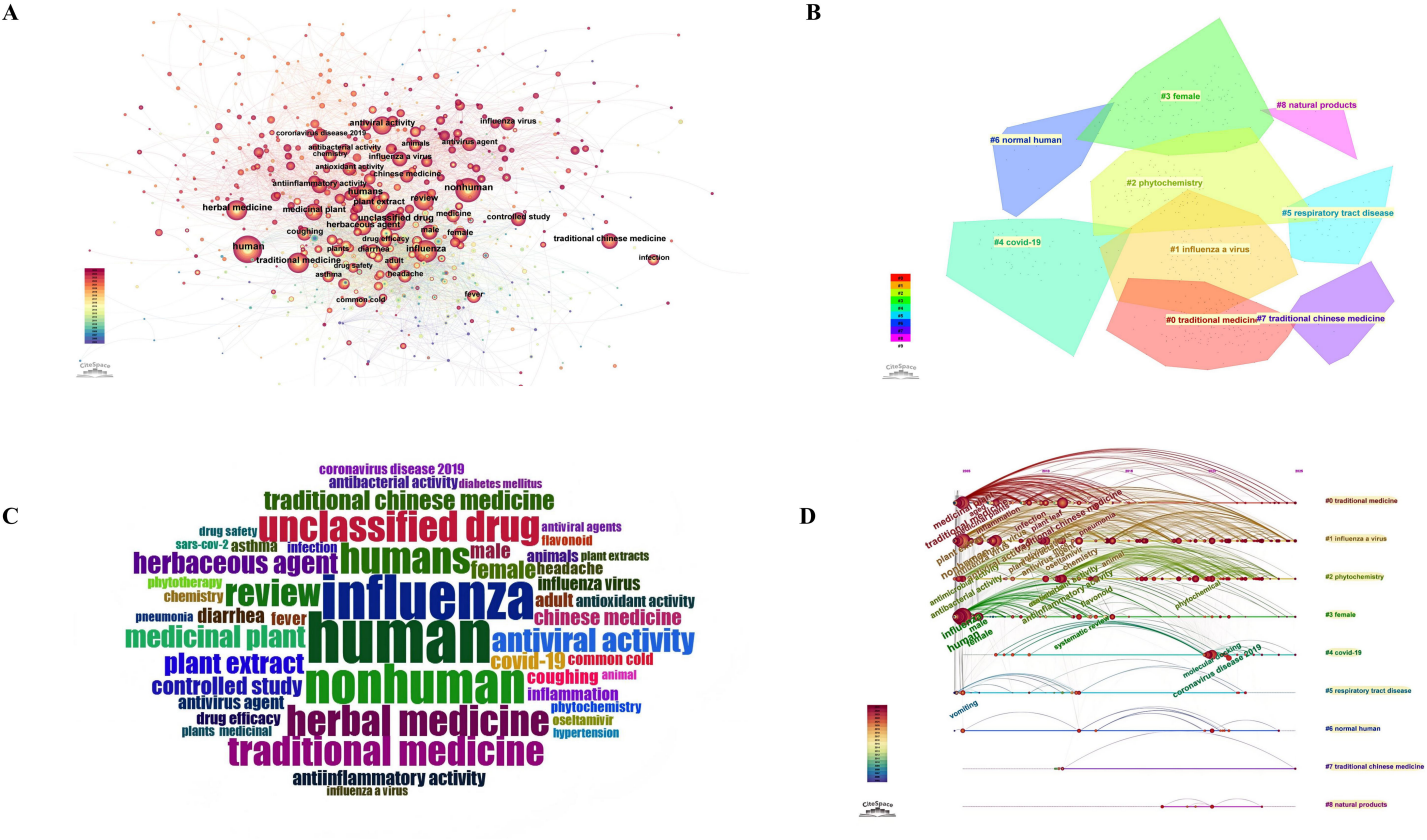
Keywords mapping of TCM for influenza treatment research. **(A)** Keywords co-occurrence network. Nodes (keywords) are sized by frequency; edges reflect co-occurrence strength, and colors show modularity-based thematic clusters. **(B)** Keyword clustering map. Colors represent different clusters, and node size indicates frequency. **(C)** Keyword cloud map. Word size reflects frequency. **(D)** Keyword timeline visualization. The horizontal axis shows publication years, with each line representing a thematic cluster labeled by a key term. Nodes within clusters indicate keywords, sized by frequency and linked via co-occurrence. Their position marks the first year of appearance, illustrating topic emergence, continuity, and development over time.

Keyword analysis reveals the breadth of research topics and their dynamic evolution in the field of Traditional Chinese Medicine for influenza treatment. Based on an analysis of 1,527 publications, a total of 3,941 author keywords were extracted. As shown in Figures 8A,C, the term “human” appeared with the highest frequency (477 times), highlighting the clinical research-oriented nature of the field. Research focus is heavily concentrated on core concepts such as “herbal medicine” (286 times), “traditional medicine” (276 times), and “traditional Chinese medicine” (179 times), with a cumulative frequency exceeding 700 times, confirming the central role of TCM in this research domain. At the same time, the high frequency of “unclassified drug” (286 times) reflects the common challenges and unique research space regarding the compositional clarity of TCM compound formulations and extracts. In terms of mechanisms of action, “antiviral activity” (222 occurrences) ranked first in both frequency and total link strength, indicating that direct antiviral effects currently represent the primary pharmacological explanatory pathway. Meanwhile, “anti-inflammatory activity” (136 occurrences), as an important complementary mechanism, collectively reflects the multi-target interventional characteristics of TCM therapy.

In Figure 8B, cluster analysis further delineates the knowledge structure of this field. Cluster #0 (traditional medicine) and Cluster #7 (traditional Chinese medicine) are associated with Cluster #3 (female) and Cluster #6 (normal human), reflecting a focus on the clinical application of traditional theories and their efficacy in human populations (33). Cluster #1 (influenza A virus) extends to Cluster #5 (respiratory tract disease), representing modern mechanism-oriented and intervention-focused research targeting specific pathogens. Cluster #8 (natural products) focuses on identifying antiviral or immunomodulatory active components from medicinal plants (34). Figure 8D clearly illustrates the thematic evolution trends. Early-stage research primarily focused on the “pathogenesis” of influenza and “clinical trials” of classical herbal formulations. After 2020, driven by the COVID-19 pandemic, the research focus in this field shifted significantly, with “COVID-19,” “SARS-CoV-2,” and “cytokine storm” rapidly emerging as hotspots. Meanwhile, “network pharmacology” and “molecular docking” continued to gain sustained attention as important methodological trends. This indicates that while deepening traditional research, the field has also responded swiftly to global public health events and promoted its own modernization through the integration of cutting-edge interdisciplinary methodologies.

## 4 Discussion

### 4.1 Research hotspots and frontiers

#### 4.1.1 Anti-influenza activity of TCM monomers

The active monomers of Traditional Chinese Medicine exert therapeutic effects against influenza through multiple pathways, including direct antiviral action, immune modulation, and symptom alleviation. For example, honeysuckle extract has been shown to inhibit the replication of influenza A virus strains H1N1, H3N2, and the oseltamivir-resistant mutant H1N1-H275Y, and is often used in the early stage of influenza (35). Modern pharmacological studies have revealed that ephedrine-alkaloid-free Ephedra Herb extract possesses c-Met inhibitory, analgesic, and anti-influenza activities, while exhibiting a better safety profile compared with conventional Ephedra Herb extract (36). Isatis indigotica polysaccharides exhibit potent anti-influenza A virus (IAV) activity against human seasonal strains (H1N1 and H3N2) and avian influenza viruses (H6N2 and H9N2) *in vitro*, and can alleviate excessive pro-inflammatory responses (37). Active monomers derived from traditional Chinese medicine, such as forsythoside A from Forsythia suspensa, baicalein from Scutellaria baicalensis, and emodin derived from Rheum officinale, have been demonstrated to directly inhibit influenza virus replication. These TCM monomers can block viral nucleoprotein export, suppress viral RNA polymerase activity, or modulate key inflammatory signaling pathways such as NF-κB and MAPK, thereby simultaneously inhibiting the virus and alleviating excessive inflammatory responses (38–41). Amygdalin, the key bioactive component in bitter almond, exhibits anti-inflammatory, anti-hypoxic, and anti-influenza virus activities, and promotes endothelial cell migration *in vitro* (42). Shikimic acid extracted from star anise, serving as a synthetic precursor of the anti-influenza drug oseltamivir, represents a prominent example of how TCM resources contribute to modern drug development (43). Research on these monomeric components provides direct lead compounds for the development of novel anti-influenza agents with well-defined structures and clear mechanisms of action.

#### 4.1.2 Therapeutic effects of TCM compounds against influenza

Unlike chemical drugs that target single pathways, the core advantage and highest form of clinical application of TCM in combating influenza lies in its compound formulations. These are not mere combinations of individual herb effects, but rather follow the compatibility theory of “sovereign, minister, assistant, and envoy.” Through the synergistic integration of multiple components, targets, and pathways, this approach enables comprehensive intervention against the influenza virus itself, the host immune response, and clinical symptoms.

In the treatment of influenza-like illness, MaXingShiGan Decoction (MXSGD) has been identified as a core prescription through data mining analysis and is widely used in clinical practice (44). Multiple studies have confirmed its efficacy in alleviating pneumonia symptoms induced by the influenza virus. In a mouse model infected with influenza A virus (IAV), MXSGD significantly improved lung injury, reduced inflammatory responses, lowered viral load, and regulated macrophage polarization (45). Additionally, MXSGD has been found to mitigate IAV-induced pneumonia by modulating the gut microbiota and reducing lipopolysaccharide levels, further suppressing glycolysis in lung tissue and balancing M1/M2 macrophage polarization, thereby exerting anti-inflammatory and immunomodulatory effects (46, 47). Both Yinqiao San (YQS) and Sangju Yin are known to exert heat-clearing and detoxifying effects. Specifically, YQS can downregulate pro-inflammatory cytokines and restore immune balance. Moreover, studies have confirmed that its 50% ethanolic extract is the most effective method for inhibiting IAV (48). Gui Zhi Ma Huang Ge Ban Tang is clinically effective for treating influenza of the wind-cold fettering the exterior pattern. It can significantly alleviate symptoms such as aversion to cold and chills, and reduce pulmonary inflammation (49). Dayuanyin has demonstrated significant efficacy in treating COVID-19 through multiple targets, multiple channels, and multiple pathways (50). Studies have also shown that Huanglian Jiedu Tang can serve as an adjuvant for H1N1 infection, and its potent active compounds may be developed as NA-1 inhibitors (51). Chaihu Guizhi Decoction exhibits therapeutic effects against depression-like symptoms induced by IAV infection by elevating dopamine and norepinephrine levels, downregulating viral M gene expression, mitigating the cytokine storm, and maintaining the homeostasis of Th17/Treg cells (52).

#### 4.1.3 Research on standardized Chinese patent medicines for influenza

Through the integration of classical compound theory and modern pharmaceutical technology, a range of proprietary Chinese medicines for influenza have become important frontline options in clinical practice. They are characterized by well-documented efficacy, controllable quality, and convenient administration, with modern research gradually elucidating their multi-component, multi-target regulatory mechanisms. For example, Lianhua Qingwen Capsule (LHQW), a widely used proprietary Chinese medicine for respiratory viral infections, has had its therapeutic effects confirmed by multiple studies (53). As an adjunctive therapy, LHQW significantly improves the clinical response and cure rates in influenza patients while reducing the incidence of adverse effects. Mechanistically, LHQW modulates signaling pathways such as TNF and TLR4, thereby enhancing survival rates, promoting weight recovery, lowering viral titers, elevating antibody levels, and alleviating lung injury. Further network pharmacology studies indicate that it acts by suppressing TLR4/MyD88 expression, regulating inflammation and oxidative stress, reducing pulmonary cell apoptosis, and demonstrating efficacy in pediatric influenza treatment (54). Additionally, it offers a potential novel strategy for the prevention of seasonal influenza in close contacts (55). Similarly, studies on Jinhua Qinggan Granules in the treatment of influenza and COVID-19 have shown that it demonstrates particular efficacy in alleviating fever symptoms. It also effectively mitigates lung inflammation and reduces the risk of pneumonia exacerbation by promoting neutrophil apoptosis and suppressing the TLR4/MyD88/NF-κB signaling pathway (56, 57). Liushen Wan has been confirmed to downregulate the expression of influenza virus-induced inflammatory factors by modulating the TLR4/NF-κB signaling pathway, thereby inhibiting viral replication *in vitro* and mitigating viral pneumonia-induced lung injury (58). The Shufeng Jiebiao formula has also been shown to alleviate excessive immune responses and thereby reduce virus-induced acute lung injury by suppressing the overactivation of the NF-κB and ERK MAPK signaling pathways (59). Analysis through multi-omics and machine learning approaches revealed that the antiviral mechanism of the Fangqin Qinggan formula is associated with the regulation of targets such as Myd88 and Ccl5, providing new target clues for anti-influenza research in TCM (60). Qingfei Dayuan Granules also demonstrate significant efficacy in treating influenza of the lung heat toxin syndrome pattern and upper respiratory tract infections (URTIs) (61, 62). Research on population-specific use of anti-influenza proprietary Chinese medicines has also advanced. A multicenter, randomized, non-inferiority trial on pediatric influenza demonstrated that Xiao’er Fengre Qing Oral Liquid (XFQOL) was non-inferior to oseltamivir in key efficacy endpoints, including time to resolution of major symptoms, time to fever relief, and improvement in symptom scores. Moreover, the safety profile showed no significant difference between the two groups, suggesting that XFQOL can serve as an alternative option for the treatment of influenza in children (63). Furthermore, the combination of syndrome differentiation-based use of proprietary Chinese medicines with standard antiviral therapy can further enhance therapeutic outcomes. For example, the Mahuang Fuzi Xixin decoction combined with oseltamivir and ribavirin significantly shortens the duration of fever and improves viral clearance rates in elderly influenza patients (64). It is worth noting that some proprietary Chinese medicines traditionally not primarily used for respiratory infections have also demonstrated anti-influenza potential. For example, Qipi Pills, mainly indicated for tonifying qi, strengthening the spleen, and promoting digestion, have shown direct antiviral efficacy against both influenza A and B viruses *in vitro* studies (65).

In summary, TCM demonstrates multi-dimensional and multi-level characteristics in combating influenza. TCM monomers exert direct antiviral effects, such as inhibiting viral replication and entry, while also possessing immunomodulatory and anti-inflammatory properties. This provides a well-defined library of lead compounds for drug development. Herbal formulations represent the core clinical practice, embodying the combinatorial principle of “sovereign, minister, assistant, and envoy” (Jun-Chen-Zuo-Shi). Through the synergistic action of multiple components, targets, and pathways, they comprehensively regulate viral infection, host inflammatory responses (e.g., via TLR/NF-κB, MAPK pathways), immune balance, and complications such as pneumonia. Proprietary Chinese medicines, based on classical formulas, offer standardized and convenient options. High-quality clinical trials and mechanistic studies have confirmed that they exhibit efficacy comparable to standard antiviral drugs in alleviating symptoms, shortening disease duration, and reducing complications, with a favorable safety profile.

### 4.2 Future perspectives

With the development of modern science and technology, research on TCM for influenza has made significant progress at multiple levels. In the areas of network pharmacology and molecular docking, the pharmacological effects of TCM compound formulations or monomeric components are revealed holistically by constructing drug-component-target-disease networks. For example, Wushen Wan has been studied through *in vivo* metabolomic profiling and network pharmacology, identifying its active constituents and elucidating its immunomodulatory activity, which was found to be enriched in the mitogen-activated protein kinase (MAPK) pathway (66). Research on the mechanisms of action of Andrographis paniculata in treating influenza, through network pharmacology and molecular docking studies, has identified its active components and targets—such as TNF, IL-6, AKT1, GAPDH, and STAT3—revealing its multi-target effects (67). Through a combination of computational and experimental approaches, the molecular mechanism of Mahuang Decoction (MHD) against influenza has been elucidated. The studies indicate that MHD targets key molecules such as AKT1, EGFR, and SRC, with critical compounds including kaempferol, quercetin, and luteolin. The underlying mechanisms involve antiviral, anti-inflammatory, and immunomodulatory activities (68). The anti-influenza potential of Eucommiae Cortex has been revealed through bioinformatics analysis combined with *in vitro* experiments. Its active compounds, such as quercetin, genistein, and kaempferol, target key molecules including IL-6, BCL-2, IL-1β, and TNF. Molecular docking further confirmed the strong binding affinity of kaempferol to TNF (69). These studies have fully leveraged the advantages of network pharmacology, providing an efficient tool for the macroscopic understanding of the mechanisms of action of Traditional Chinese Medicine and for the screening of active components. In the field of multi-omics and systems biology, a metabolomic study of Fufang Xiling Jiedu capsule in rats was conducted using ultra-high performance liquid chromatography-quadrupole time-of-flight tandem mass spectrometry (UPLC-QTOF/MS). The study identified multiple prototype components and metabolites absorbed *in vivo*, providing valuable data for subsequent pharmacodynamic, toxicological, and mechanistic investigations (70). In order to enhance the efficiency of developing anti-influenza drugs from Traditional Chinese Medicine, novel screening and evaluation technologies have been developed. For instance, a neuraminidase-based electrochemical biosensor has been created for the high-throughput screening of antiviral compounds from TCM, providing a novel strategy and technical support for establishing cost-effective, environmentally friendly, and rapid screening platforms (71). Furthermore, technical guidelines have been established for the non-clinical pharmacological research of TCM compound formulations. These guidelines aim to standardize investigations, enhance research quality, and facilitate the translation of basic research into clinical application. They emphasize the integration of multidimensional qualitative pharmacokinetic and pharmacodynamic studies with quantitative “PK-PD” research on key components, in order to build a complete evidence chain for the efficacy of TCM compounds (72). These methodological innovations provide more efficient and precise tools for anti-influenza research in Traditional Chinese Medicine, thereby helping to advance its standardization and modernization. However, it should be noted that these methods yield largely predictive or exploratory evidence. Their reliability is constrained by incomplete databases, algorithmic variability, and limited standardization. Crucially, the lack of *in vivo* validation, pharmacodynamic support, and clinical correlation means these findings cannot yet be equated with definitive therapeutic evidence.

Future TCM anti-influenza research will trend toward technological advancement and systematic integration. Yet, priority must be given to evidence quality over technical application. This requires bridging computational predictions with *in vitro*/*in vivo* validation, integrating multi-omics data, and establishing a rigorous evidence chain through standardized clinical studies. Such efforts are essential to systematically decode the scientific basis of TCM’s efficacy against influenza.

### 4.3 Limitations

Although existing studies have made some progress in the clinical efficacy and mechanisms of TCM for influenza, several limitations remain. First, bibliometric analysis mainly reveals research hotspots, collaboration networks, and development trends, but cannot directly assess study quality, evidence level, or risk of bias. Therefore, the findings should be further validated by systematic reviews or meta-analyses. Second, TCM is characterized by “multi-component, multi-target, and multi-pathway” effects, making its mechanism network highly complex. Most current mechanistic studies are still based on *in vitro* experiments or network pharmacology predictions and focus on single pathways or a few targets, which makes it difficult to fully explain the overall effects of Chinese herbal formulas and multi-component synergy. Meanwhile, the *in vivo* metabolism, bioavailability, and precise interactions between active compounds and key targets require further investigation. In addition, there is substantial heterogeneity in formula composition, dosage, intervention duration, and outcome measures across studies, which limits the comparability and generalizability of the findings.

This study has certain limitations. First, the bibliometric analysis was based on WOSCC, Scopus, and PubMed and included only English publications, which may introduce language and database bias. As these databases mainly index English-language journals, important Chinese studies, including high-impact domestic literature, may have been underrepresented. Future studies should integrat Chinese databases such as CNKI and Wanfang to improve comprehensiveness and representativeness. Second, bibliometrics mainly depends on quantitative indicators such as publication counts and citation frequencies, which do not directly assess the intrinsic quality, clinical relevance, or methodological rigor of the research. Consequently, some highly cited but controversial studies may be overemphasized, while high-quality recent work that has not yet accumulated sufficient citations may be undervalued. Therefore, future research should incorporate multilingual databases, introduce qualitative evaluation dimensions, and pay close attention to the latest scientific developments to more comprehensively and balancedly reveal the evolving trajectory and knowledge structure of this field.

## 5 Conclusion

Current evidence indicates that TCM exerts anti-influenza effects through multi-component, multi-target, and multi-pathway mechanisms. Monomers exhibit direct antiviral and anti-inflammatory activities, serving as promising lead compounds. Classical formulas, guided by compatibility theory, regulate immunity and reduce lung injury, reflecting systemic therapeutic advantages. Proprietary Chinese medicines demonstrate clinical efficacy comparable to oseltamivir in relieving symptoms and shortening disease duration, with favorable usability. Future studies should prioritize integrating high-quality clinical trials with mechanistic research, clarifying the material basis and signaling pathways of TCM, and advancing international standardization and translational applications.

## Data Availability

The original contributions presented in this study are included in this article/supplementary material, further inquiries can be directed to the corresponding authors.

## Footnotes

1
https://www.bibliometrix.org
